# Antibiotic prescribing and outcomes in cancer patients with febrile neutropenia in the emergency department

**DOI:** 10.1371/journal.pone.0229828

**Published:** 2020-02-28

**Authors:** Olivier Peyrony, Camille Gerlier, Imola Barla, Sami Ellouze, Léa Legay, Elie Azoulay, Sylvie Chevret, Jean-Paul Fontaine

**Affiliations:** 1 Emergency Department, Hôpital Saint-Louis, Paris, France; 2 Emergency Department, Hôpital Saint-Joseph, Paris, France; 3 Intensive Care Unit, Hôpital Saint-Louis, Paris, France; 4 Centre de Recherche en Épidémiologie et Statistiques, Université de Paris (CRESS-INSERM-UMR1153), Epidemiology and Clinical Statistics for Tumor, Respiratory, and Resuscitation Assessments (ECSTRRA) Team, Paris, France; 5 Université de Paris, Paris, France; 6 Biostatistics and Medical Information Department, Hôpital Saint-Louis, Paris, France; Fundacao Oswaldo Cruz, BRAZIL

## Abstract

**Introduction:**

The benefit of reducing the time of antibiotic initiation in the emergency department (ED) for neutropenic patients is controversial and the research on the impact of antibiotic adherence to international guidelines in the ED is scarce. We aimed to investigate the effect of antibiotic timing and appropriateness on outcomes in patients with febrile neutropenia (FN) and to assess the performance of the MASCC risk-index to risk-stratify such patients in the ED.

**Methods:**

We prospectively identified patients with FN who presented to our ED and assessed their Multinational Association of Supportive Care in Cancer (MASCC) risk-index. The time to parenteral antibiotic initiation and the appropriateness of the antibiotic regimen according to international guidelines were retrospectively abstracted. The performance of the MASCC risk-index in predicting the absence of complication was assessed with sensitivity, specificity and the area under the receiver operating characteristics curve (AUC). We investigated the effect of the time to antibiotic initiation and the appropriateness of the antibiotic regimen on the outcome (ICU admission or death) by logistic regression analyses.

**Results:**

We included 249 patients. Median age was 60 years and 67.9% had hematological malignancies, 26 (10.4%) were admitted to the ICU and 23 (9.8%) died during hospital stay. Among the 173 patients at low risk according to the MASCC risk-index, 56 (32.4%) presented at least one complication including 11 deaths. The MASCC risk-index had a sensitivity and a specificity of 0.78% and 0.43%, respectively, in predicting the absence of complication and the AUC was 0.67. The time to antibiotic initiation in the ED was not associated with the outcome after adjusting for performance status and shock-index. Conversely, an inadequate ED antibiotic regimen was associated with higher ICU admission or death during hospital stay (OR = 3.50; 95% CI = 1.49 to 8.28).

**Conclusion:**

An inadequate ED antibiotic regimen in patients with FN was significantly associated with higher ICU admission or death during hospital stay.

## Introduction

Cancer is of growing interest in the emergency literature. Three years ago, experts in the field of oncology and urgent care identified research priorities among cancer emergencies [1]. Febrile neutropenia was one of these topics of prime interest, particularly the identification of optimal treatment strategies, including the effects of the timing of initial antibiotic therapy, biomarkers and early risk-stratification tools. The benefit of reducing the time of antibiotic initiation in the emergency department (ED) for neutropenic patients is controversial [2–7]. Moreover, research on the impact of the antibiotic adherence to international guidelines [8, 9] in the ED is scarce [10]. Another major challenge in febrile neutropenic patients in the ED is to identify patients at low-risk of worsening and eligible to be safely discharged home with oral antibiotics. Very few tools exist to risk-stratify neutropenic patients and the Multinational Association of Supportive Care in Cancer (MASCC) risk-index is one of them [11]. This tool is widely used in the oncology field and is recommended by international guidelines [8] but has shown some limits in the emergency setting [12]. With this study performed in the ED, we aimed to investigate the effect of antibiotic timing and appropriateness on outcomes in patients with febrile neutropenia and to assess the performance of the MASCC risk-index in risk-stratifying such patients in the ED.

## Material and methods

### Ethical aproval

The study was approved by the “Comité d’Evaluation de l’Ethique des projets de Recherche Biomédicale (CEERB) Paris Nord” (Institutional Review Board -IRB 00006477- of HUPNVS, Paris 7 University, AP-HP)—number 2019–008. All data were fully anonymized

### Objectives

The objective of this study was to identify predictors of intensive care unit (ICU) admission or death in cancer patients with febrile neutropenia consulting an ED. More particularly, the role of the time of antibiotic initiation and of the appropriateness of the antibiotic regimen according to international guidelines was evaluated. Lastly, the performance of the MASCC risk-index in predicting the risk of complications in febrile neutropenic patients in the ED was assessed.

### Patients, setting and study design

The study took place between January 2016 and December 2017 in the ED of Saint-Louis hospital (Paris, France). This university hospital has 650 beds, including 350 beds dedicated to curing malignancies. The ED receives 41,000 patients per year, including 15% of patients with malignancies. Our ED has a short hospitalization unit that is used as a step-down unit or an observation location for holding to see if patients need hospitalization. The medical ICU is a closed 12-bed unit that admits 600 patients per year, including about 130 cancer patients. ICU admission is considered when a cancer patient has at least one organ failure or presents with physiological derangements that foreshadow organ dysfunction. Early ICU admission is strongly encouraged to improve survival by preventing the development or progression of organ dysfunctions. During the study period, we prospectively identified patients with febrile neutropenia who presented to our ED and assessed their MASCC risk-index with a dedicated form. Patients were included if they were older than 15 years and had febrile neutropenia defined according to the Infectious Disease Society of America (IDSA) [8]. Patients who presented several times to our ED for the same episode of neutropenic fever were included only once, at the first presentation. Then, we retrospectively abstracted medical records to collect data that are reported in tables and figures. These data included patient age, sex, underlying malignancy, performance status (0: fully active, able to carry on all pre-disease performance without restriction, 1: Restricted in physically strenuous activity but ambulatory and able to carry out work of a light or sedentary nature, 2: Ambulatory and capable of all self-care but unable to carry out any work activities up and about more than 50% of waking hours, 3: Capable of only limited self-care, confined to bed or chair more than 50% of waking hours, 4: Completely disabled, cannot carry on any self-care, totally confined to bed or chair; poor performance status was defined as 3 or 4), significant comorbidities, time since the beginning of fever, prior antibiotic treatment before the ED visit, clinical findings at the ED presentation (signs of sepsis or septic shock, clinical focus of infection, diarrhea, mucositis) and vital signs at triage including shock-index (heart rate/systolic blood pressure), serum biological tests (creatinine, lactate, hemoglobin), antibiotic regimen administered in the ED and the time between first medical contact and antibiotic administration, results from microbiological investigations and classification of neutropenic fever as fever of unknown origin (FUO), clinically documented infection, and microbiologically documented infection, length of hospital stay, occurrence of complications during hospital stay or soon after hospital discharge, ICU admission during hospital stay and status (dead or alive) at hospital discharge and at day 90.

### Definitions

#### Antibiotic appropriateness

According to the IDSA guidelines [8], the 4^th^ European Conference on Infections in Leukemia [9] and our hospital microbial ecology, the initial antibiotic regimen initiated in the ED was considered as adequate if this regimen included:

- an association of amoxicillin-clavulanic acid and a fluoroquinolone (ofloxacin, ciprofloxacin or levofloxacin) or a monotherapy with parenteral cephalosporin (cefotaxime or ceftriaxone) for low-risk patients according to the MASCC risk-index;
- a monotherapy with an antipseudomonal beta-lactam (piperacillin-tazobactam, cefepim, axepim or ceftazidime) or with imipenem-cilastatin in case of prior colonization or infection with extended-spectrum beta-lactamase (ESBL) producing bacteria for high-risk patients without sepsis or septic shock;
- the adjunction of an aminoglycoside in case of sepsis or septic shock;
- the adjunction of vancomycin in case of skin or central venous catheter (CVC) infection or pneumonia or prior colonization with methicillin-resistant *staphylococcus aureus* (MRSA) in patients with sepsis or septic shock.

The antibiotic regimen was considered as inadequate if there was:

- no antipseudomonal beta-lactam in case of high-risk patient according to the MASCC risk-index;
- no aminoglycoside in case of sepsis or septic shock;
- no vancomycin in case of skin or CVC infection or mucositis or pneumonia in patients with sepsis or septic shock;
- no imipenem-cilastatin in case of prior colonization or infection with ESBL-producing bacteria.

#### Complications for MASCC accuracy evaluation

Briefly, the MASCC risk-index is a score that was validated by Klastersky et al. It ranges from 0 to 26 depending on outpatient status, age, burden of illness, hypotension, dehydration, history of chronic obstructive pulmonary disease, the type of the malignancy (solid or hematological) and history of fungal infection. A score ≥ 21 indicates the patient is at low risk of complication (<10%). Complications were defined by the authors as follows: Death during hospital stay, systolic blood pressure < 90 mmHg or need for vasopressor support to maintain blood pressure, arterial oxygen pressure ≤ 60 mmHg while breathing on room air or need for mechanical ventilation, intensive care unit admission, disseminated intravascular coagulation, confusion or altered mental state, congestive cardiac failure seen on chest x-ray and requiring treatment, bleeding severe enough to require transfusion, arrhythmia or ECG changes requiring treatment, renal failure requiring investigation and/or treatment with intravenous fluids, dialysis, or any other intervention, and other complications judged serious and clinically significant. For non-admitted patients after ED visit, any re-hospitalization for persistent fever in the next 5 days was considered as a complication. In our study, we used the same definition for complications as those listed by Klastersky et al.

#### Outcome for ED risk factor identification

The outcome was a composite outcome, including ICU admission or death during hospital stay.

### Statistical analysis

Results are reported as median with interquartile range (IQR) for continuous variables and number with percentage for binary and categorical variables. Time was used as a continuous variable. Patient characteristics were compared using the chi square test for categorical variables and the Wilcoxon Mann Whitney test for continuous variables. Accuracy of the MASCC risk-index in predicting the absence of complication was assessed with a receiver operating characteristics (ROC) curve and by calculating the area under the curve (AUC). Sensitivity, specificity, and positive and negative predictive values were calculated for the validated threshold of 21.

We investigated predictive factors of the outcome (i.e., ICU admission or death) using logistic regression analyses. Four patients who had received prior parenteral antibiotics in another care center were excluded. Five patients who did not receive parenteral antibiotics in the ED and were discharged with oral antibiotics were also excluded from the analysis. Univariable models were first fitted. Then, in order to consider potential confounders, we fitted multivariable models including variables with clinical significance based on literature or expert opinion, which were associated with the outcome on the basis of *p*-values less than 0.1 by univariable analyses. Then, we applied a backward selection procedure based on *p*-values but also on clinical significance, favoring variables that reflected patient severity, such as shock-index, and functional status, such as performance status. As the timing of antibiotic administration and the appropriateness of the antibiotic regimen were the exposures of interest, these variables were forced into the multivariable logistic model.

Missing data were managed with multiple imputation by chained equations [13]. As recommended [14], variables included in the imputation model were those of the logistic regression prediction model (including the outcome), in addition to auxiliary covariates correlated with the missing variables; 30 datasets were imputed with 50 iterations each. The multivariable logistic regression model was applied to the 30 imputed datasets and final estimates were obtained by averaging the 30 estimates according to Rubin’s rules.

As a sensitivity analysis, we plotted the Kaplan-Meier curve of the ICU free survival (from ED presentation up to 90 days) according to the appropriateness of the antibiotic regimen. In this analysis, for patients who visited the ED several times for distinct episodes of febrile neutropenia, only the first visit was included. Survival curves were compared using the log-rank test.

All *p*-values were two-sided, with values of 0.05 or less considered as statistically significant.

Data were analyzed with R 3.5.0 software (the R Foundation for Statistical Computing, Vienna, Austria).

## Results

### General characteristics

During the study period, 249 patients were included. Median age was 60 years and 67.9% had hematological malignancies. Lymphoma, acute leukemia and breast tumor were the most frequent cancers. Malignancies were controlled in 70 (28.1%) of the cases and patients had good performance status in 87.3% of the cases. Before visiting the ED, fever lasted for 1 day (median) and 31.7% of patients had already begun oral antibiotics at home prescribed systematically by their oncologist or hematologist in case of fever. In most of the cases there was an association of amoxicillin-clavulanic acid and a fluoroquinolone. In the ED, 60.6% of patients had clinical focus of infection, mostly pulmonary. Patients had signs of sepsis or septic shock in 17.3% of the cases and a high shock-index at triage in 37.1% of the cases (Table 1).

**Table 1:** Patient characteristics.

Characteristics (N = 249)			Missing data
**Age, median [IQR], years**	60	[43–71]	0
**Female gender, n (%)**	141	(56.6)	0
**Underlying malignancy, n (%)**			0
Hematological	169	(67.9)	
Lymphoma	67	(26.9)	
Acute leukemia	59	(23.7)	
Myelodysplastic syndrome	11	(4.4)	
Myeloma	11	(4.4)	
Chronic leukemia	6	(2.4)	
Other	15	(6.0)	
Solid	80	(32.1)	
Breast	52	(20.9)	
Lung	9	(3.6)	
Urinary tract	8	(3.2)	
Digestive	4	(1.6)	
Other	7	(2.8)	
**Time since diagnosis, n (%)**			1
Diagnosis	9	(3.6)	
< 1 year	141	(56.9)	
1 to 5 years	58	(23.4)	
> 5 years	40	(16.1)	
**Chemotherapy line, median [IQR]**	1	[1–2]	2
**Evolution, n (%)**			0
Newly diagnosed	74	(29.7)	
Controlled	70	(28.1)	
Progression	105	(42.2)	
**Performance status, n (%)**			12
Good (≤2)	207	(87.3)	
Poor (>2)	30	(12.7)	
**Palliative status, n (%)**	32	(12.9)	0
**Cardiovascular diseases, n (%)**	69	(27.7)	0
**Prophylaxis with fluoroquinolones, n (%)**	4	(1.6)	0
**Diabetes mellitus, n (%)**	23	(9.2)	0
**Long course steroids (> 1 month), n (%)**	35	(14.1)	0
**HIV infection, n (%)**	11	(4.4)	0
**Bone marrow transplant, n (%)**	16	(6.4)	0
**Immunosuppressive agent, n (%)**	8	(3.2)	0
**Days of fever before ED, median [IQR]**	1	[1–2]	9
**Previous oral antibiotics before ED, n (%)**	79	(31.7)	0
**Diarrhea, n (%)**	49	(19.7)	0
**Mucositis, n (%)**	57	(22.9)	0
**Clinical focus of infection in the ED, n (%)**			0
None	98	(39.4)	
Pulmonary	55	(22.1)	
Skin/Mucositis/CVC	37	(14.9)	
Digestive	36	(14.4)	
ENT	15	(6.0)	
Urinary	6	(2.4)	
Other	2	(0.8)	
**Sepsis or shock, n (%)**	43	(17.3)	0
**MASCC risk-index**			
Median [IQR]	22	[19–24]	0
High risk, N (%)	76	(30.5)	
Low risk, N (%)	173	(69.5)	
**High shock-index (≥1), n (%)**	92	(37.1)	1

*CVC* central venous catheter, *ED* emergency department, *HIV* human immunodeficiency virus, *IQR* interquartile range, *MASCC* Multinational Association of Supportive Care in Cancer, *ENT* ear-nose-throat

### Parenteral antibiotics in the ED

Two hundred sixteen (86.7%) patients received an anti-pseudomonal beta-lactam, 34 (13.6%) an aminoglycoside, 24 (9.6%) vancomycin and 8 (3.2%) a carbapenem. The antibiotic regimen was inadequate for 53 (21.3%) patients. Reasons for the inadequacy of the regimen included the absence of an aminoglycoside adjunction in case of hemodynamic instability (37.8%), absence of imipenem-cilastatin in case of prior colonization or infection with ESBL-producing bacteria (32.1%), absence of vancomycin in case of hemodynamic instability and pneumonia or mucositis or skin infection (20.7%) and absence of an antipseudomonal betalactam in case of high-risk patient (9.4%). Median time of parenteral antibiotic initiation in the ED was 90 [45–150] minutes. Infection was microbiologically documented in 89 (35.7%) cases, clinically documented in 46 (18.5%) cases, and fever was of unknown origin in 114 (45.8%) cases. Details of microbiological and clinical documentations are given in the S1 Table.

### Performance of the MASCC risk-index

**Fig 1:**
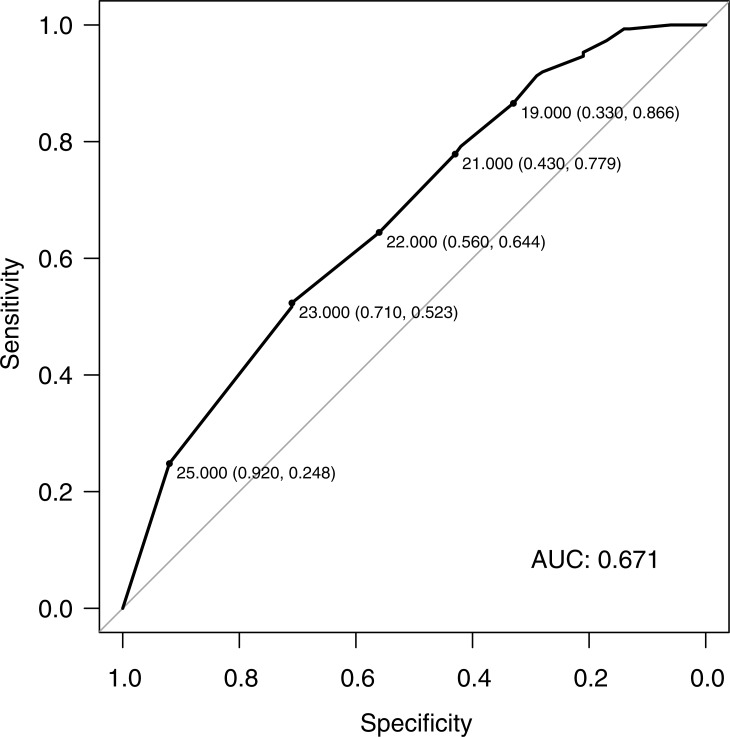
Receiver operating characteristics curve. Accuracy of the MASCC risk-index in predicting the absence of complication for neutropenic patients in the ED. Specificity and sensitivity are indicated for each threshold. *AUC* area under the curve, *MASCC* Multinational Association of Supportive Care in Cancer.

Fig 1 shows the accuracy of the MASCC risk-index in predicting the absence of complication at different thresholds (AUC = 0.67; 95% CI: 0.60 to 0.74). At the conventional threshold of 21, the MASCC risk-index had a sensitivity, a specificity, and a positive and a negative predictive value of 0.78%, 0.43%, 67.6% and 56.6% respectively, with significantly fewer complications in low-risk compared to high-risk patients (*p* = 0.0006), Table 2. Among the 173 low-risk patients, 56 (32.4%) presented at least one complication, including 11 in-hospital deaths. Details of the complications are shown in the S2 Table.

**Table 2:** Complications according to the MASCC risk-index.

MASCC risk-index	No complication (n = 150)		Complication (n = 99)		*p*
**Low risk, ≥21 (N = 173), n (%)**	117	(67.6)	56	(32.4)	0.0006
**High risk, <21 (N = 76), n (%)**	33	(43.4)	43	(56.6)	

*MASCC* Multinational Association of Supportive Care in Cancer

### Outcome

Patients were mostly hospitalized after the ED visit (94.4%). Among these patients, 26 (10.4%) were admitted to the ICU, 23 (9.8%) died during hospital stay and 7 died in ICU (Table 3). Thus, 42 (16.9%) patients were admitted to the ICU or died during hospital stay.

**Fig 2:**
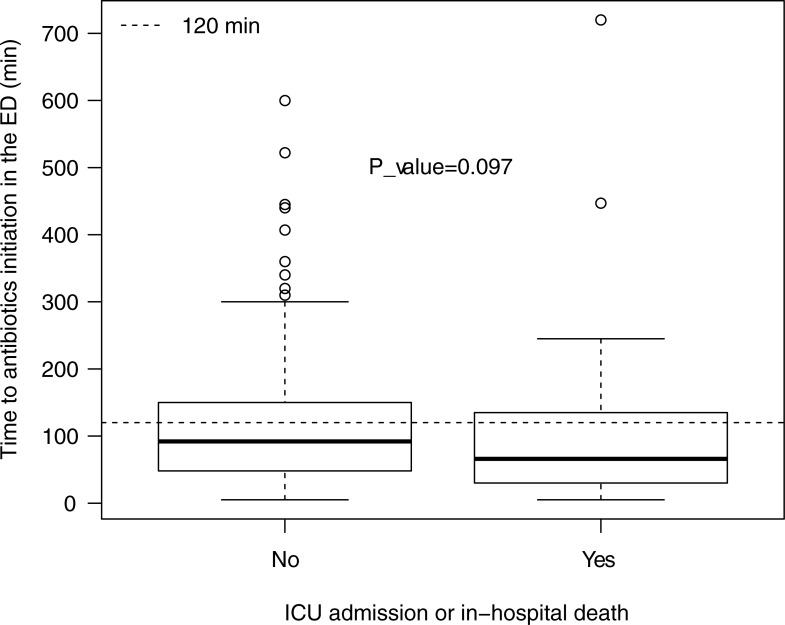
Boxplot. Time to antibiotics initiation in the ED depending on the outcome (ICU or death during hospital stay). Median and IQR were 92 [48–150] min for those who did not meet the outcome and 66 [30–135] min for those who did with no statistical difference according to the Wilcoxon Mann Whitney test (*p* = 0.097).

**Fig 3:**
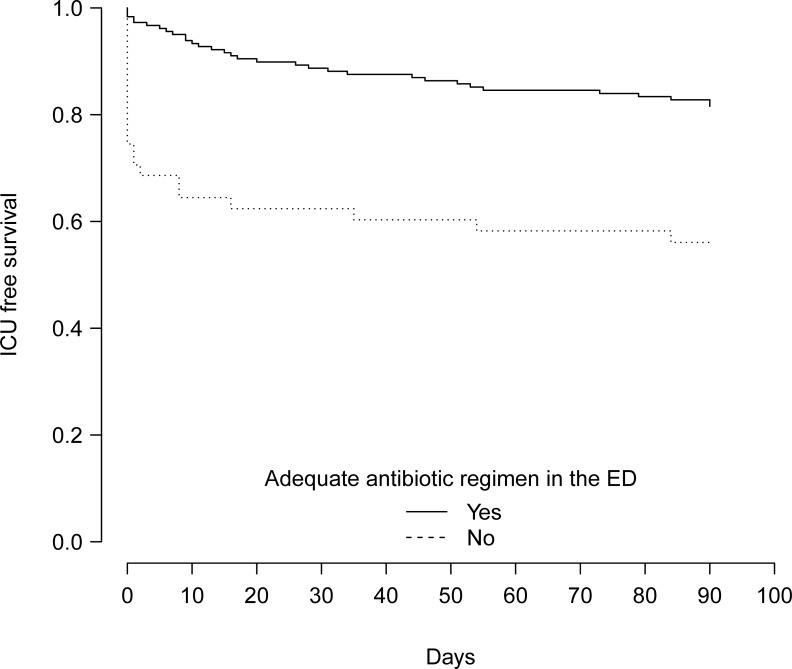
Kaplan-Meier curves of the ICU free survival during 90 days from ED presentation depending on the appropriateness of the antibiotic regimen initiated in the ED.

The median time to parenteral antibiotics initiation in the ED was 66 [30–135] min for those who were admitted to the ICU or died during hospital stay and 92 [48–150] min for those who did not (*p* = 0.097), Fig 2. Results of univariable analyses are shown in the S3 Table. In the multivariable analysis (Table 4), after multiple imputation, time to antibiotic initiation in the ED was not associated with the outcome (OR = 1.00/min; 95% CI = 1.00 to 1.00, *p* = 0.7), after adjusting for performance status and shock-index. Conversely, an inadequate ED antibiotic regimen was associated with higher ICU admission or death during hospital stay (OR = 3.50; 95% CI = 1.49 to 8.28, *p* = 0.004). ICU free survival was significantly improved in patients who received an adequate antibiotic regimen in the ED (Fig 3), *p* = 0.00001).

**Table 3:** Patient outcomes after ED.

Characteristics (N = 249)			Missing data
**Post ED orientation, n (%)**			0
Discharged home	14	(5.6)	
ED hospitalization unit	122	(49.0)	
ICU	16	(6.4)	
Wards	97	(39.0)	
**Complication or death during hospital stay or soon after discharge, n (%)**	99	(39.8)	0
**Among hospitalized patients (n = 235)**			
**ICU during hospital stay, n (%)**	26	(10.4)	0
**Time to ICU admission, median [IQR], days**	0	[0–1]	0
**Time to first complication, median [IQR], days**	2	[1–5]	1
**Death during hospital stay, n (%)**	23	(9.8)	0
**Time to in-hospital death, median [IQR], days**	9	[4–16]	0
**Length of hospital stay, median [IQR], days**	7	[4–12]	0
**ICU or death during hospital stay, n (%)**	42	(16.9)	0
**Location after hospital discharge, n (%)**			0
Discharged home	189	(80.4)	
Nursing home or palliative care	23	(9.8)	
Deceased	23	(9.8)	

*ED* emergency department, *IQR* interquartile range

**Table 4:** Multivariable analysis. Variables independently associated with ICU admission or in-hospital mortality.

Variable	Model without imputation (n = 228)			Model with imputation (n = 240)		
	aOR	95% CI	*p*	aOR	95% CI	*p*
**Poor performance status (>2)**	8.89	[3.47 to 23.58]	<0.00001	7.19	[2.86 to 18.09]	0.00004
**High shock-index (≥1)**	2.53	[1.14 to 5.73]	0.02	2.65	[1.20 to 5.85]	0.02
**Inadequate ED antibiotics**	3.50	[1.49 to 8.28]	0.004	3.65	[1.62 to 8.25]	0.002
**Time to ED antibiotics initiation (/min)**	1.00	[1.00 to 1.00]	0.7	1.00	[1.00 to 1.00]	0.8

*aOR* adjusted Odds Ratio; *ED* emergency department

## Discussion

This study performed in the ED appraises outcomes and targets for improving management in patients with febrile neutropenia. The finding that inappropriate antibiotic prescribing was associated with higher ICU admission or mortality rates stresses the need to improve the skills of ED specialists treating this high-risk population. Moreover, our study did not demonstrate that timing of antibiotic initiation in the ED was associated with poor outcomes. Thus, even if initiating antibiotics promptly is recommended in emergency situations such as neutropenic fever, initiating too quickly may be harmful if this precipitance hampers the choice of the adequate antibiotics. This assumption is in line with the concerns recently published by the IDSA about the last Surviving Sepsis Campaign Guidelines [15] where the authors stated that administration of IV antimicrobials should be initiated within one hour for both sepsis and septic shock [16]. Although the IDSA agreed that antimicrobials should be initiated as soon as possible in patients with severe infections, they were more cautious about fixing a time period and feared that this straightjacket approach impels caregivers to administer broad-spectrum antibiotics to uninfected patients with sepsis-like syndromes [15]. In the same way, the European Society of Emergency Medicine considered that completion of a sepsis bundle within 1 hour after triage which was not evidence-based may be unrealistic and potentially harmful [17].

These concerns may apply to the neutropenic patient who is considered infected when he becomes febrile, even though fever is of unknown origin most of the time. Obviously, in the absence of neutrophils, the site of infection may be lacking, and thus infection must be presumed and antibiotics started. But, the benefit of reducing the timing of antimicrobial initiation is uncertain. While some studies showed that shortening the time of antibiotic administration significantly reduced the length of hospital stay [2, 3], serious complications [4], ICU admission and 30-day mortality [7, 18], others failed to demonstrate any benefit with regard to the length of hospital stay [5] or serious complication [6]. In patients admitted to the ICU with severe sepsis or septic shock, mortality was higher when antimicrobial initiation exceeded 1 hour after the first sign of sepsis [19] or 2 hours after ICU admission [20]. Nonetheless, despite these conflicting results, the IDSA recommended initiating antibiotics within 120 minutes of presentation in patients with febrile neutropenia [8] and reduced this delay to 60 minutes in their last update [21], increasing the likelihood that broad-spectrum antibiotics will be given more frequently without collecting clinical and laboratory data, and, more importantly, without looking for prior infections or colonization with MDR pathogens.

In a study that included 25,231 patients admitted with febrile neutropenia, Wright et al. showed that among low-risk patients, use of guideline-based antibiotics reduced the risk of in-hospital mortality [10]. The adjunction of aminoglycosside in case of hemodynamic instability has been suggested, but remains controversial. Legrand et al. showed that combination antibiotic therapy which includes an aminoglycoside was associated with lower mortality in a cohort of 428 neutropenic patients admitted to the ICU for severe sepsis or septic shock [22]. Even though this association between appropriateness of initial antibiotic regimen and mortality needs to be confirmed in large prospective studies, the rationale remains strong. For instance, extending antibiotic spectrum using bactericidial drugs in this population at risk for MDR pathogens is certainly of interest [23]. Moreover, as about half the patients exhibited FUO, it is difficult to apply guidelines that may have been issued for given organ infections. Finally, as the vast majority of patients were further hospitalized, it is likely that these patients were the sickest and the challenge was more about infection control.

In our multivariable logistic regression model, a poor performance status and a high shock-index were associated with ICU admission or in-hospital death. These two variables have already been studied and associated with poor outcome in cancer patients [7, 23–26] and non-cancer patients [27, 28]. If shock-index is not specific to cancer patients, its use at the ED triage is very simple and permits early recognition of patients at high risk of complication, as does the quick Sequential Organ Failure Assessment score [29].

Klastersky et al. first validated the MASCC risk-index in 2000 and found that complications occurred in less than 10% of low-risk patients [11]. Other authors found similar results with a very low rate of complications in low-risk patients [30, 31]. It is important to underscore that these studies included mostly patients with solid malignancies and lymphomas. In other studies with a higher prevalence of hematological malignancies, complications occurred in more than 15% of these low-risk patients [32, 33]. Moreover, we believe that febrile neutropenic patients attending the ED are sicker, increasing the likelihood that complications occur, than those in oncology wards or out of the hospital when the fever starts. Therefore, the positive predictive value of the MASCC risk-index is lower in this population and false positives (complications in the low-risk group) are higher. Recently, the MASCC risk-index was studied in patients with febrile neutropenia in the emergency setting [12]. In this study, 16% of the low-risk patients had complications. Thus, thus MASCC risk-index may be inaccurate in distinguishing patients at low risk in the ED, and even more so when the prevalence of hematological malignancies is high.

### Limitations

The retrospective methodology of our study certainly introduced some interpretation bias during medical record abstraction. Nevertheless, in order to avoid misinterpretations, the MASCC risk-index was prospectively collected, and we selected objective adjusting variables (shock-index instead of sepsis or septic shock, and performance status instead of cancer evolution or palliative status) and variables of interest such as time to antibiotics or type of antibiotics that were clearly identified in the ED medical records. Moreover, it has been shown that reliability is higher for studies based on explicit criteria and that focused on outcome rather than process errors [34]. Antibiotic appropriateness may be more questionable and might vary from one center to another. However, we defined it according to international guidelines and to the strength of these recommendations. The number of covariates introduced in the multivariate analysis was limited by the low prevalence of the outcome and it is possible that some potential confounders were missed in the analysis. However, the composite outcome (ICU admission and death) appeared more reliable than serious complications, the clinical significance of which may vary from one physician to another. Furthermore, a more robust methodology, such as randomizing antibiotic accurateness or time to antibiotics initiation, is obviously not possible. The results of this single-center study inevitably require external validation. Whether these results actually apply to low-volume centers remains to be determined.

## Conclusions

In this study which included 249 patients with febrile neutropenia presenting to the ED, time to antibiotic initiation was not significantly associated with outcomes. However, an inadequate ED antibiotic regimen was significantly associated with higher ICU admission or death during hospital stay. Studies to improve antibiotic prescribing in ED patients with febrile neutropenia are warranted.

## Supporting information

S1 Table Neutropenic fever classification and microbiological and clinical documentations. (PDF)

S2 Table Complications in low-risk patients according to the MASCC risk-index. (PDF)

S3 Table Univariable analysis. Variables associated with in -hospital ICU admission or death
(PDF)

## Abbreviations

AUC: area under the curve
CVC: central venous catheter
ECIL: European Conference on Infections in Leukemia
ED: emergency department
ESBL: extended-spectrum beta-lactamase
FUO: fever of unknown origin (FUO)
ICU: intensive care unit
IDSA: Infectious Disease Society of America
IQR: interquartile range
MASCC: Multinational Association of Supportive Care in Cancer
MRSA: methicillin-resistant *staphylococcus aureus*
ROC: receiver operating characteristic
