# Supplementary material for: Antibiotic prescribing and outcomes in cancer patients with febrile neutropenia in the emergency department
Source: PLoS One. 2020 Feb 28;15(2):e0229828. doi: 10.1371/journal.pone.0229828 (PMC7048306; doi:10.1371/journal.pone.0229828)
Supplement: S1 Table — (PDF) [file pone.0229828.s001.pdf]

S1 Table Neutropenic fever classification and microbiological and clinical documentations

Documentation (N=249), n (%)		
**Microbiologically documented infection**	89	(35.7)
**Isolated pathogens (n=107)**		
**Bacteria**	80	(74.8)
**Gram negative**	49	(61.2)
Escherichia coli	25	(51.0)
Pseudomonas aeruginosa	9	(18.4)
Klebsiella	5	(10.2)
ESBL producing bacteria	11	(22.4)
**Gram positive**	30	(37.5)
Clostridium difficile	12	(40.0)
Staphylococcus	8	(26.7)
Streptococcus	5	(16.7)
Methicillin resistant bacteria	2	(7.0)
**Virus**	21	(19.6)
Influenza A/B	6	(28.6)
Herpes simplex virus	3	(14.3)
Syncytial respiratory virus	2	(9.5)
**Fungal**	5	(4.7)
Aspergillus	3	(60.0)
Candida albicans	2	(40.0)
**Parasite**	1	(0.9)
Plasmodium malariae	1	(100.0)
**Clinically documented infection**	46	(18.5)
Pulmonary	17	(37.0)
Skin	12	(26.1)
Digestive	9	(19.6)
ENT	7	(15.2)
Joint	1	(2.1)
**Fever of unknown origin**	114	(45.8)

ENT ear-nose-throat, *ESBL* extended-spectrum beta-lactamase
