# Supplementary material for: Antibiotic prescribing and outcomes in cancer patients with febrile neutropenia in the emergency department
Source: PLoS One. 2020 Feb 28;15(2):e0229828. doi: 10.1371/journal.pone.0229828 (PMC7048306; doi:10.1371/journal.pone.0229828)
Supplement: S2 Table — (PDF) [file pone.0229828.s002.pdf]

S2 Table Complications in low-risk patients according to the MASCC risk-index

Complications in low-risk patients (n=56)		
**Death, n (%)**	11	(19.3)
**Renal failure, n (%)**	5	(8.8)
**Intensive care unit admission, n (%)**	4	(7.0)
**Shock, n (%)**	3	(5.2)
**Respiratory failure, n (%)**	3	(5.2)
**Severe bleeding, n (%)**	2	(3.5)
**Arrhythmia, n (%)**	2	(3.5)
**Confusion or altered mental state, n (%)**	1	(1.8)
**Disseminated intravascular coagulation, n (%)**	1	(1.8)
**Congestive cardiac failure, n (%)**	1	(1.8)
**Other complications judged serious and clinically significant by the investigator, n (%)**	23	(42.1)
Cytopenia requiring transfusion	7	(12.3)
Severe colitis	3	(5.2)
Severe mucositis	3	(5.2)
Prolonged fever	3	(5.2)
Anaphylaxis	2	(3.5)
Catheter withdrawal	2	(3.5)
Hepatitis	1	(1.8)
Varicella zoster virus infection	1	(1.8)
Graft versus host disease	1	(1.8)
Hospitalization after ED discharge	1	(1.8)

ED emergency department, *MASCC* Multinational Association of Supportive Care in Cancer
