# Supplementary material for: Antibiotic prescribing and outcomes in cancer patients with febrile neutropenia in the emergency department
Source: PLoS One. 2020 Feb 28;15(2):e0229828. doi: 10.1371/journal.pone.0229828 (PMC7048306; doi:10.1371/journal.pone.0229828)
Supplement: S3 Table — Variables associated with in -hospital ICU admission or death (PDF) [file pone.0229828.s003.pdf]

S3 Table Univariable analysis. Variables associated with in -hospital ICU admission or death

Variable	ICU admission or death during hospital stay		OR	95% CI	*p*	Missing data
	No (n=207)	Yes (n=42)				
**Age, median [IQR], years**	56 [40-70]	67 [59-73]	1.03	(1.01 to 1.06)	0.002	0
**Sex (male)**	83 (40.1)	25 (59.5)	2.20	(1.12 to 4.38)	0.02	0
**Underlying malignancy, n (%)**						0
Hematological malignancy	138 (66.7)	31 (73.8)	1			
Solid malignancy	69 (33.3)	11 (26.2)	0.71	(0.32 to 1.46)	0.4	
**Time since diagnosis, n (%)**						1
Diagnosis	8 (3.9)	1 (2.4)	1			
< 1 year	119 (57.8)	22 (52.4)	1.48	(0.25 to 28.13)	0.7	
1 to 5 years	48 (23.3)	10 (23.8)	1.67	(0.26 to 32.69)	0.6	
> 5 years	31 (15.0)	9 (21.4)	2.32	(0.35 to 46.03)	0.5	
**Chemotherapy line, median [IQR]**	1 [1-2]	1 [1-2]	1.02	(0.81 to 1.27)	0.8	2
**Evolution, n (%)**						0
Newly diagnosed or Controlled	129 (62.3)	15 (35.7)	1			
Progression	78 (37.7)	27 (64.3)	2.98	(1.51 to 6.07)	0.002	
**Poor performance status (>2), n (%)**	16 (8.1)	14 (35.0)	6.09	(2.65 to 14.01)	0.00002	12
**Palliative status, n (%)**	18 (8.7)	14 (33.3)	5.25	(2.33 to 11.75)	0.00005	
**Cardiovascular disease, n (%)**	51 (24.6)	18 (42.9)	2.29	(1.14 to 4.56)	0.02	0
**Diabetes mellitus, n (%)**	15 (7.2)	8 (19.0)	3.01	(1.14 to 7.51)	0.02	0
**Long course steroids, n (%)**	24 (11.6)	11 (26.2)	2.71	(1.17 to 5.99)	0.02	0
**HIV infection, n (%)**	9 (4.3)	2 (4.76)	1.10	(0.16 to 4.47)	0.9	0
**Bone marrow transplant, n (%)**	9 (4.3)	7 (16.7)	4.40	(1.49 to 12.59)	0.006	0
**Immunosuppressive agent, n (%)**	6 (2.9)	2 (4.8)	1.68	(0.24 to 7.57)	0.5	0
**Days of fever before ED, median [IQR]**	1 [1-2]	1 [1-2]	0.94	(0.71 to 1.20)	0.6	9
**Previous oral antibiotics before ED, n (%)**	67 (32.4)	12 (28.6)	0.84	(0.39 to 1.70)	0.6	0
**Diarrhea, n (%)**	38 (18.4)	11 (26.2)	1.58	(0.70 to 3.35)	0.2	0
**Mucositis, n (%)**	48 (23.2)	9 (21.4)	0.90	(0.38 to 1.95)	0.8	0
**Clinical focus of infection in the ED, n (%)**	119 (57.5)	32 (76.2)	2.37	(1.14 to 5.31)	0.03	0
**Sepsis or shock, n (%)**	19 (9.2)	24 (57.1)	13.19	(6.18 to 29.13)	<0.00001	0
**High shock-index (≥1), n (%)**	65 (31.6)	27 (64.3)	3.90	(1.97 to 8.00)	0.0001	1
**Oxygen saturation, median [IQR]**	99 [98-100]	97 [97-99]	0.80	(0.69 to 0.91)	0.001	1
**Inadequate ED antibiotics, n (%)**	33 (15.9)	20 (47.6)	4.79	(2.35 to 9.81)	0.00002	0
**Time to antibiotics in the ED, median [IQR], min**	92 [48-150]	66 [30-135	1.00	(1.00 to 1.00)	0.097	9
**MASCC risk-index, median [IQR]**	22 [21-24]	17 [11-21]	0.77	(0.71 to 0.84)	<0.00001	0

ED emergency department, HIV human immunodeficiency virus, IQR interquartile range, ICU intensive care unit, MASCC Multinational Association of Supportive Care in Cancer
